# Happiness inequality has a Kuznets-style relation with economic growth in China

**DOI:** 10.1038/s41598-022-19881-3

**Published:** 2022-09-20

**Authors:** Pan Zhang

**Affiliations:** 1 School of International and Public Affairs, Shanghai Jiao Tong University, 1954# Huashan Road, Xuhui District, Shanghai, 200030 China; 2 China Institute for Urban Governance, Shanghai Jiao Tong University, 1954# Huashan Road, Xuhui District, Shanghai, 200030 China

**Keywords:** Psychology, Health care

## Abstract

Happiness studies generally investigate average levels of happiness rather than happiness inequality between regions, and studies of social inequality usually measure it based on the distribution of life opportunities (e.g., income) rather than life results (e.g., happiness). Inspired by the Kuznets curve, which illustrates the inverted U-shaped correlation between income inequality and economic growth, this study investigates whether there is a subjective wellbeing Kuznets curve. It uses data from ten waves of the Chinese General Social Survey to construct a panel data set and runs panel data models to investigate the hypothesized curvilinear relationship between happiness inequality and economic growth. The results show that happiness inequality, measured as the standard deviations of respondents’ self-reported happiness, first increases and then decreases as per-capita GDP increases in Chinese provinces. These findings strongly support the subjective wellbeing Kuznets curve hypothesis and suggest that strategies for reducing happiness inequality must consider stages of economic development.

## Introduction

Social inequality is a practical and theoretical problem that is central to the question of how to build an egalitarian society^1^. For example, there is much evidence that income inequality is a breeding ground for social problems such as poor health, violence, drug abuse, and lack of trust^2,3^. Some scholars argue that the distribution of income does not capture all aspects of social inequality^4^, although previous studies focus on income inequality across countries and regions^1,5,6^. This can be attributed to the limitation that income is a measure of life opportunities, which are preconditions for high quality of life, but not the measure of life results^7,8^. Veenhoven^7^ divides life results into external life results related to utility of life and internal life results related to satisfaction with life. Subjective wellbeing, which is a pervasive sense of how good one’s life is^9^, can be a good proxy for life results. Thus, it is critical to investigate how subjective wellbeing is distributed across regions and why.

In the field of income inequality, Kuznets predicts that income inequality first increases, then remains steady, and finally decreases as the economy grows^10^. Dozens of empirical studies following Kuznets’s work confirm the inverted U-shaped effect of economic growth on income inequality, which is now known as the Kuznets curve^5^. Inspired by the Kuznets curve hypothesis, some scholars hypothesize and confirm similar relationships between economic growth and other phenomena, including environmental degradation^11–13^, gender inequality^14^, and crime^15^. However, no studies explore whether there is a subjective wellbeing Kuznets curve. Although some scholars argue that income inequality and inequality of subjective wellbeing are based on the distribution of life opportunities and life results respectively^7^, both measures are used to examine social inequality. Accordingly, one way to advance research on the inequality of subjective wellbeing is to draw on the research on income inequality.

Consequently, this study examines whether there is a Kuznets curve for the inequality of subjective wellbeing. In other words, it tests whether the correlation between economic growth and the inequality of subjective wellbeing is an inverted U-shape. In academic research, subjective wellbeing is usually measured in terms of happiness or life satisfaction^16,17^. Following previous research, this study uses ten waves of the Chinese General Social Survey (CGSS) to develop a panel data set in which the inequality of subjective wellbeing is measured using the standard deviations in respondents’ perceived happiness in each Chinese province. A set of panel data models indicates that the dispersion of respondents’ self-reported happiness first increases and then decreases as per-capita GDP grows, supporting the existence of a subjective wellbeing Kuznets curve. In addition, the estimation shows that happiness inequality reaches a turning point when per-capita GDP reaches about 32,577.17 Chinese yuan based on unchanged 2006 prices. Among the 25 Chinese provinces considered in this study, about two thirds have passed the turning point, such as Tianjin, Shanghai, Beijing, Jiangsu, and Zhejiang. These findings imply that to reduce happiness inequality, it is necessary to promote economic development and adjust policy priorities according to a region’s economic development stage.

## Review of research on happiness inequality

Happiness is a person’s subjective perception or feeling of her life-as-a-whole^18^. Levels of happiness are used to measure the overall happiness of people in a region, and happiness inequality focuses on variations in people’s happiness in a region—that is, the degree of divergence in the level of happiness^19^. High happiness inequality means that happiness is more unequally distributed among the population, while low happiness inequality means that the distribution of happiness is more equal. When happiness inequality is high, an individual’s happiness level is different from the levels of others. To develop a good measure for happiness inequality, Kalmijn and Veenhoven^20^ use eight criteria to evaluate nine kinds of inequality indicators and conclude that four statistical indicators can produce satisfactory results: standard deviation, interquartile range, mean pair difference, and mean absolute difference. In view of the widespread use of standard deviation, they also encourage researchers to continue to use standard deviation to measure happiness inequality^20^.

The few studies on happiness inequality can be mainly divided into two categories. The first explores the antecedents of happiness inequality at the micro level and usually uses the recentered influence function (RIF) regression to run data at the individual level^21,22^. For example, Yang, Liu, and Zhang^22^ use the RIF regression to examine the impacts of demographic characteristics on happiness inequality in China. Using an RIF regression estimation, Niimi^23^ finds that household income, people’s perceptions of their relative income rank, job insecurity, and post-retirement life affect happiness inequality in Japan. Becchetti, Massari, and Naticchioni^21^ also use the RIF regression method to confirm that income, education, and unemployment are important drivers of happiness inequality in Germany. However, as Niimi^23^ notes, studies in this cluster use cross-sectional data to explore the determinants of happiness inequality but do not apply panel data analysis.

The second cluster investigates happiness inequality using panel data from various regions or countries at the macro level^4,17^. Some scholars examine trends in happiness inequality. For example, Dutta and Foster^24^ find that happiness inequality in the USA began to decrease in the 1970s and did not begin to rise until the 2000s, whereas Veenhoven^7^ finds that the dispersion of life satisfaction in EU nations shrank during the 1973–2001 period. In addition, some studies explore how happiness inequality is correlated with institutional factors using correlation analysis^25–27^. They consider institutional factors such as social security, governance quality, economic freedom, and government size^25–27^. Other studies investigate what influences regional happiness inequality by running panel data regression models. For example, Bennett and Nikolaev^1^ examine how economic freedom influences happiness inequality, and Ovaska and Takashima^4^ considers the effects of political freedom, economic freedom, health inequality, and income inequality on happiness inequality.

To advance research on happiness inequality, in 2005, the *Journal of Happiness Studies* published a special issue on the problems related to the study of happiness inequality, ranging from its measurement to its trends and determinants^19^. Although these efforts have encouraged scholars to devote more attention to happiness inequality, studies of happiness inequality are still much less common than studies of average levels of happiness^1,18,24^. In summary, there are still many gaps in the understanding of happiness inequality. First, although research on happiness inequality draws on research on income inequality, many factors known to be related to income inequality and theories that explain income inequality have not been transferred to studies of happiness inequality. Second, most studies on determinants of happiness inequality focus on identifying linear correlations and do not explore complex relationships, especially curvilinear relationships. Third, current data on happiness inequality are mainly from developed countries, and more evidence from developing countries is needed.

## Nexus between economic growth and happiness inequality

If a subjective wellbeing Kuznets curve exists, happiness inequality will present an inverted U-shaped relation with economic growth. In other words, happiness inequality will first increase and then decrease as the economy grows. There are at least three mechanisms through which economic growth affects happiness inequality. First, it can do so through the distribution of household income. Generally, the Kuznets curve hypothesis holds that the impact of economic growth on income inequality has an inverted U-shape, which means that the dispersion of income first increases and then decreases in relation to economic growth^5,10^. In addition, many studies find that income inequality is positively correlated with happiness inequality^4,8^, confirming that the dispersion of happiness increases as the dispersion of income increases. Considering these findings together, it is reasonable to hypothesize that the correlation between happiness inequality and economic growth has an inverted U-shape.

Second, good governance may be a mechanism through which economic growth influences happiness inequality. The accumulation of economic wealth will increase citizens’ demands for good governance. For example, many countries have tried to encourage innovation to improve government efficiency, strengthen accountability, amplify citizens’ voices, improve regulation quality, and promote the rule of law^28–31^. In addition, previous studies confirm that the technical quality of government (measured in terms of government effectiveness, regulatory quality, rule of law, and control of corruption) has an inverted U-shaped relation with happiness inequality^26^. The relationship between the democratic quality of government (measured in terms of voice and accountability, and political stability) and happiness inequality also has an inverted U-shape^27^. Because economic growth breeds high quality of government and the latter has an inverted U-shaped relation with happiness inequality, the correlation between happiness inequality and economic growth is expected to have an inverted U-shape.

Third, economic growth can influence happiness inequality through environmental pollution, which is regarded as an important determinant of happiness^13,17,32,33^. Specifically, according to the environmental Kuznets curve, environmental pollution first become worse and then becomes better as the economy grows^11,12^. Some studies find that poorer households tend to purchase masks and air filters to alleviate the negative influence of environmental pollution, whereas wealthier people tend to invest more in air filters that offer stronger protection^34^. This means that wealthier people have more capital and a stronger capability of mitigating the negative effects of environmental pollution on happiness. As wealthier people generally have higher initial levels of happiness than those with less wealth^35^, the imbalance between wealthier and less wealthy persons in their ability to mitigate air pollution can aggravate happiness inequality when environmental pollution increases. Taking these arguments together, the hypothesis is proposed:

Hypothesis 1: Happiness inequality has an inverted U-shaped relation with economic growth such that happiness inequality first increases and then decreases along with economic growth.

## Methodology

### Empirical strategy

This study uses a province-level panel data set from China to explore whether there is a subjective wellbeing Kuznets curve in China. Building on the Kuznets curve hypotheses in different policy domains (e.g., the Kuznets curve in income inequality and the environmental Kuznets curve)^5,10–12^, this study estimates panel data models that consider GDP per capita together with its quadratic terms. The empirical model is as follows:

$${\text{HIE}}_{{{\text{i}},{\text{t}}}} = {\upgamma }_{0} + {\upgamma }_{1} {\text{GDP}}_{{{\text{i}},{\text{t}} - 1}} + {\upgamma }_{2} {\text{GDP}}_{{{\text{i}},{\text{t}} - 1}}^{2} + {\upgamma }_{3} {\text{Control}}_{{{\text{i}},{\text{t}} - 1}} + \upvarepsilon_{{{\text{i}},{\text{t}}}}$$.

In this equation, ${\text{HIE}}_{{{\text{i}},{\text{t}}}} { }$ denotes happiness inequality in province *i* in year *t*. ${\text{GDP}}_{{{\text{i}},{\text{t}} - 1}}$ denotes the GDP per capita of province *i* in year *t* − 1, which is used to reflect economic growth, and ${\text{ GDP}}_{{{\text{i}},{\text{t}} - 1}}^{2}$ denotes its quadratic term, which is the most important variable for determining whether a subjective wellbeing Kuznets curve exists. ${\text{Control}}_{{{\text{i}},{\text{t}} - 1}} { }$ denotes a vector consisting of all controls and $\upvarepsilon_{{{\text{i}},{\text{t}}}}$ is the disturbance term. ${\upgamma }_{0}$ is the constant coefficient, and ${\upgamma }_{1}$_,_ ${\upgamma }_{2}$, and ${\upgamma }_{3}$ denote the regression coefficients of these variables. ${\upgamma }_{1}$ and ${\upgamma }_{2}$ can be used to judge whether a subjective wellbeing Kuznets curve exists. Specifically,

1. If ${\upgamma }_{2}$ > 0, happiness inequality has a U-shaped relation with economic growth (a convex curve).
2. If ${\upgamma }_{2}$ < 0, happiness inequality has an inverted U-shaped relation with economic growth (a concave curve); in this condition, if ${\upgamma }_{1}$ > 0, the relationship is consistent with the subjective wellbeing Kuznets curve hypothesis and the turning point can be calculated as ${ }\frac{{{-}{\upgamma }_{1} }}{{2{\upgamma }_{2} }}$ (in this case, $\frac{{{-}{\upgamma }_{1} }}{{2{\upgamma }_{2} }} > 0$).
3. If ${\upgamma }_{2}$ = 0 and ${\upgamma }_{1}$ ≠ 0, happiness inequality has a linear relation with economic growth.
4. If ${\upgamma }_{2}$ = 0 and ${\upgamma }_{1}$ = 0, no quadratic or linear relation is found between happiness inequality and economic growth.

### Data

The data describing happiness are drawn from ten waves of the CGSS conducted in 2003, 2005, 2006, 2008, 2010, 2011, 2012, 2013, 2015, and 2017. The CGSS uses a multi-stage stratified probability proportionate-to-size sampling principle^17^. An average of 10,000 households located in more than 100 counties and districts in 31 provinces in mainland China were randomly sampled in each wave of the CGSS. Survey themes vary from people’s demographic characteristics to social values and behaviors. The CGSS has become the most important source for investigating social change in China and been widely used in academic research published in international journals that undergo strict blind peer review^17,36,37^. As these surveys disclose information about each respondent’s province-level residential address, it is possible to aggregate the information to the provincial level.

Happiness is measured with one question: “Altogether, how happy do you feel about your life?” This question is analogous to the question used in the World Values Survey^38^. The surveyed individuals are asked to rate their happiness on a 5-point Likert scale ranging from 1 (very unhappy) to 5 (very happy). The standard deviation in the respondents’ self-rated happiness in each province is then calculated to give an indicator of the happiness inequality of that province^4,17,26,27^. Although all 31 provinces in mainland China were investigated during at least one wave of the CGSS, data for six provinces in mainland China (Inner Mongolia, Xinjiang, Qinghai, Ningxia, Tibet, and Hainan) are missing in at least one wave. Thus, this study uses data from the 25 provinces in the estimation that are not missing any happiness inequality.

To control for the possible effects of other factors on happiness inequality in Chinese provinces, this study follows the literature in controlling for four clusters of control variables^17^. First, happiness inequality is likely to be related to the general level of happiness^25^. Hence, the level of happiness of each investigated province is controlled; it is measured as the mean value of the happiness ratings of each province in each wave of the CGSS^17^. Second, some economic factors may also be related to happiness inequality^23,39^. Hence, this study uses the ratio of industry output, consumer price index (CPI), ratio of urban unemployed labor, and ratio of total volume of export and import trade in GDP as controls. Third, the population of each province and its demographic structure are potential factors in happiness inequality^22,40^. Therefore, the total population of each province and a set of demographic variables are also controlled, including the ratio of elderly persons, the gender ratio, and the ratio of persons who have a college degree or above. Fourth, the time trend of happiness inequality in Chinese provinces is controlled with a series of dummy variables denoting each year. Because each wave of CGSS is usually conducted at midyear, all control variables and independent variables lag for 1 year to avoid potential reverse relationship. Table 1 provides detailed information on the variables.

**Table 1:** Measures of variables.

Variable	Measure	Source
Inequality_h	Standard deviation of respondents’ self-rated happiness by province during each wave of the CGSS	CGSS
Level_h	Mean of respondents’ self-rated happiness by province during each wave of the CGSS	CGSS
Industry	Secondary industry output divided by GDP (1 year lagged, %)	CSY
Unemployed	Ratio of urban unemployed labor (1 year lagged, %)	CSY
Male	Number of males per 100 females (1 year lagged)	CSY
CPI	Consumer price index (CPI) (1 year lagged)	CSY
Aging	Ratio of persons aged 65 or above (1 year lagged, %)	CSY
Education	Ratio of persons having a college diploma or above (1 year lagged, %)	CSY
Trade	Total volume of exports and imports divided by GDP (1 year lagged)	CSY
Population	Number of total population (1 year lagged, ten thousands of persons, ln)	CSY
GDP	GDP per capita based on unchanged 2006 prices (1 year lagged, yuan/person, ln)	CSY
GDP^2^	Quadratic term of the natural logarithm of GDP per capita based on unchanged 2006 prices (1 year lagged)	CSY

*CSY* China Statistical Yearbooks. Ten waves of CGSS were used^41^, which were conducted in 2003, 2005, 2006, 2008, 2010, 2011, 2012, 2013, 2015, and 2017. In addition, 10 years of CSY were used^42^, which were published in 2003, 2005, 2006, 2008, 2010, 2011, 2012, 2013, 2015, and 2017.

## Results

### Distribution of happiness inequality and the average levels of happiness

**Figure 1:**
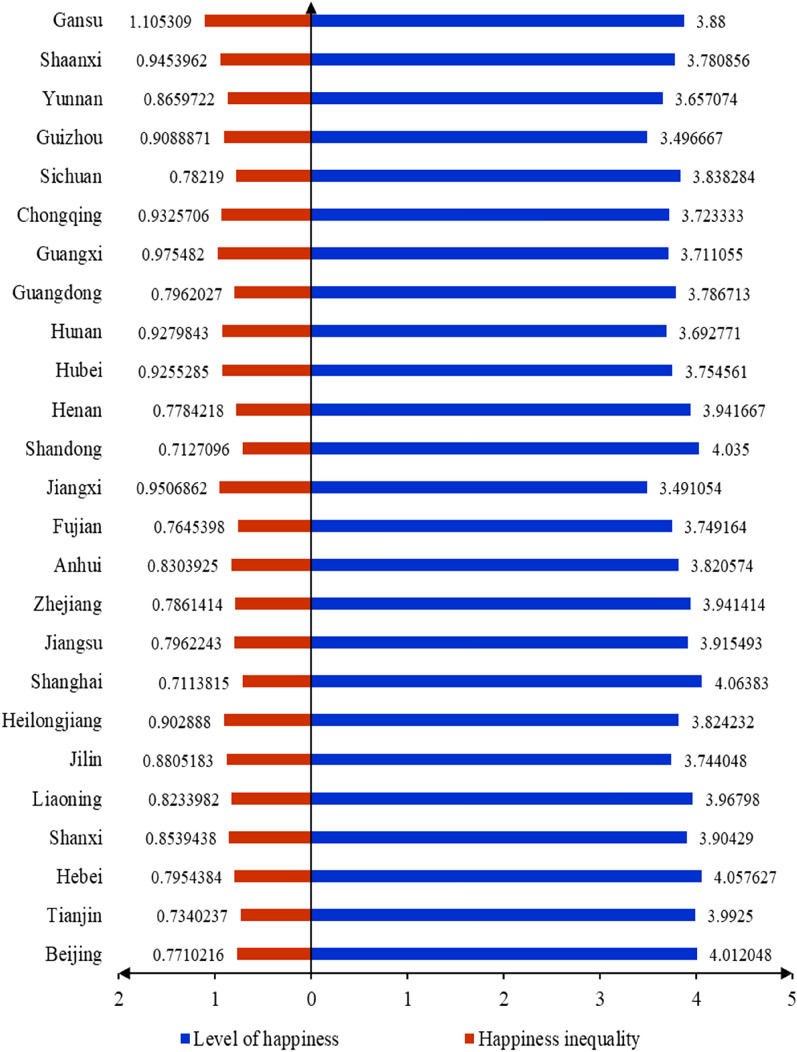
Distribution of average levels of happiness and happiness inequality in 2017^41^.

Figure 1 presents the distribution of the average levels of happiness and happiness inequality in 2017. Provinces with the highest levels of happiness do not necessarily have the highest or lowest levels of happiness inequality. For example, Shanghai, Hebei, Shandong, and Beijing have the highest levels of happiness, whereas Gansu, Guangxi, and Jiangxi have relatively high happiness inequality. Moreover, Shanghai, Shandong, and Tianjin have the three lowest levels of happiness inequality, but the average levels of happiness in Shanghai and Shandong are also high. As the average level of happiness and happiness inequality do not necessarily co-vary, efforts to increase happiness must consider how happiness inequality varies across regions.

### Descriptive and correlation analysis

The descriptive statics and bivariate correlations are presented in Tables 2 and 3, respectively. The mean, minimum, and maximum values of happiness inequality are 0.8274, 0.5765, and 1.2904 respectively, indicating high variance in happiness inequality in these provinces. This is a suitable context for exploring what determines happiness inequality. In addition, the mean of the average levels of happiness is 3.6675, meaning that the average level of happiness is moderate. In addition, the minimum value of the average level of happiness is 2.8333, around the rank of being slightly unhappy, while its maximum value is 4.3158, around the rank of being happy. This means that the average level of happiness also shows high variance across Chinese provinces. Moreover, from the maximum and minimum value of each control variable, it can be seen that the range of Education seems larger than those of the other control variables. The detailed information of happiness inequality and GDP per capita can be found in Supplementary Figs. S1 and S2 online.

**Table 2:** Descriptive Analysis.

	Obs	Mean	Std. Dev	Min	Max
Inequality_h	250	0.8274	0.1071	0.5765	1.2904
Level_h	250	3.6675	0.2714	2.8333	4.3158
Industry	250	47.4535	7.1572	19.3	60
Unemployed	250	3.608	0.7182	1.3	6.5
Male	250	104.1385	3.509	95.59	118.62
CPI	250	102.424	2.0379	97.6538	106.4058
Aging	250	9.492	1.7009	6.2984	15.3994
Education	250	9.6267	6.685	1.9941	45.462
Trade	250	0.3368	0.3792	0.0294	1.6668
Population	250	8.3914	0.5312	6.9147	9.3056
GDP	250	10.0386	0.6769	8.2713	11.5408
GDP^2^	250	101.2301	13.5699	68.415	133.1897

**Table 3:** Bivariate correlation results.

	1	2	3	4	5	6	7	8	9	10	11
1. Inequality_h	1										
2. Level_h	0.110*	1									
3. Industry	− 0.019	0.093	1								
4. Unemployed	0.044	− 0.314***	0.248***	1							
5. Male	0.082	− 0.029	− 0.193***	− 0.235***	1						
6. CPI	0.145**	0.208***	0.161**	− 0.015	0.045	1					
7. Aging	− 0.045	0.270***	− 0.083	0.071	− 0.361***	− 0.069	1				
8. Education	− 0.134**	0.393***	− 0.479***	− 0.483***	− 0.013	0.008	0.370***	1			
9. Trade	− 0.246***	0.051	0.043	− 0.191***	− 0.114*	− 0.006	0.181***	0.391***	1		
10. Population	− 0.104	0.044	0.288***	0.080	0.047	0.070	− 0.086	− 0.490***	− 0.131**	1	
11. GDP	− 0.104	0.623***	− 0.048	− 0.398***	− 0.069	0.053	0.487***	0.771***	0.530***	− 0.183***	1
12. GDP^2^	− 0.114*	0.616***	− 0.067	− 0.402***	− 0.062	0.048	0.484***	0.785***	0.535***	− 0.193***	0.999***

***p < 0.01, **p < 0.05, and *p < 0.1.

The correlation coefficients between most of the control variables are smaller than 0.6, implying that there are no strong interactions between the control variables. However, the correlation coefficient between GDP and Level_h is 0.623 (p < 0.01) and the correlation between GDP and Education is 0.771 (p < 0.01), implying strong correlations. Thus, it is necessary to put GDP per capita and its quadratic square into regressions first to control possible multicollinearity, which may result from interactions between GDP and other controls. The correlation coefficient between happiness inequality and the quadratic square of GDP per capita is − 0.114, which is significant (p < 0.1). This is in accordance with the theoretical prediction. However, because of the panel data structure, it is difficult to determine whether a subjective wellbeing Kuznets curve exists based on the bivariate correlations. Therefore, several panel data regression models are run to test the hypothesis.

### Regression analysis

Two regression models based on the full sample are presented in Table 4 (see Models 1 and 2). Only GDP per capita and its quadratic square are included in Model 1, whereas the other four clusters of control variables are included in Model 2. A Hausman test is performed to choose the fixed-effects (FE) model or the random-effects (RE) model^43^. The Hausman test for Model 1 rejects the null hypothesis, and thus, the FE model is more appropriate (Chi^2^ = 25.56, p < 0.01). Hence, this study estimates Model 1 using the FE model. However, the Hausman test for Model 2 supports the null hypothesis (Chi^2^ = 18.71, p > 0.1). Therefore, this study estimates Model 2 using the RE model. Nonetheless, the empirical results are relatively similar when both the FE model and the RE model are applied to estimate each regression model.

**Table 4:** Regression results.

	The full sample		Extreme observations excluded	
	Model 1	Model 2	Model 3	Model 4
GDP	0.9989242*** (0.2465893)	0.7834748*** (0.297563)	0.8795884*** (0.2102023)	0.6117173*** (0.233566)
GDP^2^	− 0.0480651*** (0.0122197)	− 0.0424626*** (0.0157002)	− 0.0420849*** (0.0104118)	− 0.0333748*** (0.0120703)
Level_h		− 0.0580324 (0.0454868)		− 0.0734397 (0.0393616)
Industry		− 0.0016693 (0.0012414)		− 0.0009291 (0.0011725)
Unemployed		0.000572 (0.0120336)		0.0078589 (0.0077161)
Male		− 0.0017856 (0.00156)		− 0.0011711 (0.0015343)
CPI		0.0062468 (0.0104195)		− 0.0012704 (0.0081949)
Aging		− 0.003656 (0.0037169)		− 0.0027963 (0.0034061)
Education		− 0.0022457 (0.0021108)		− 0.0010052 (0.0019218)
Trade		0.0297173 (0.0270938)		0.0191634 (0.0182496)
Population		− 0.06051*** (0.0164074)		− 0.0425534*** (0.0100012)
Constant	− 4.334773*** (1.241569)	− 2.429735 (2.119451)	− 3.749163*** (1.058624)	− 1.115897 (1.617127)
Year fixed effects		Controlled		Controlled
Observations	250	250	245	245
Hausman	25.56***	18.71	28.91***	13.29
Model	FE model	RE model	FE model	RE model
R^2^	0.1029	0.5061	0.1042	0.4862

Robust standard errors are in parentheses. ***p < 0.01, **p < 0.05. Within R^2^ is reported for the FE model, and overall R^2^ is reported for the RE model.

The regression coefficient of GDP in Model 1 is positive and significant (0.9989242, p < 0.01), whereas the regression coefficient of its quadratic square is negative and significant (− 0.0480651, p < 0.01). In relation to the discussion above, these regression results mean that happiness inequality presents an inverted U-shaped relation with GDP per capita, which supports the hypothesis of a subjective wellbeing Kuznets curve. When all of the control variables are put into Model 2, the coefficient of GDP per capita remains significantly positive (0.7834748, p < 0.01) and the coefficient of its quadratic square remains significantly negative (− 0.0424626, p < 0.01). Thus, the subjective wellbeing Kuznets curve hypothesis is also supported in Model 2. Together, these results show that happiness inequality in Chinese provinces first increases and then decreases as GDP per capita increases.

It is possible that outliers may influence the quadratic estimation results, so this study examines the robustness of these findings by dropping extreme observations. To overcome potential estimation bias, five extreme observations with happiness inequality values above 1.1 are removed from the sample and the estimation is performed again using the remaining observations (see Models 3 and 4). After these five extreme observations are removed, the coefficients of GDP per capita in Models 3 and 4 are still positive (0.8795884 and 0.6117173), whereas the regression coefficients of the quadratic square of GDP per capita are still negative (− 0.0420849 and − 0.0333748). All four of these coefficients remain statistically significant (p < 0.01). These results thus support the subjective wellbeing Kuznets curve hypothesis and strongly confirm the robustness of the empirical findings in this study.

**Figure 2:**
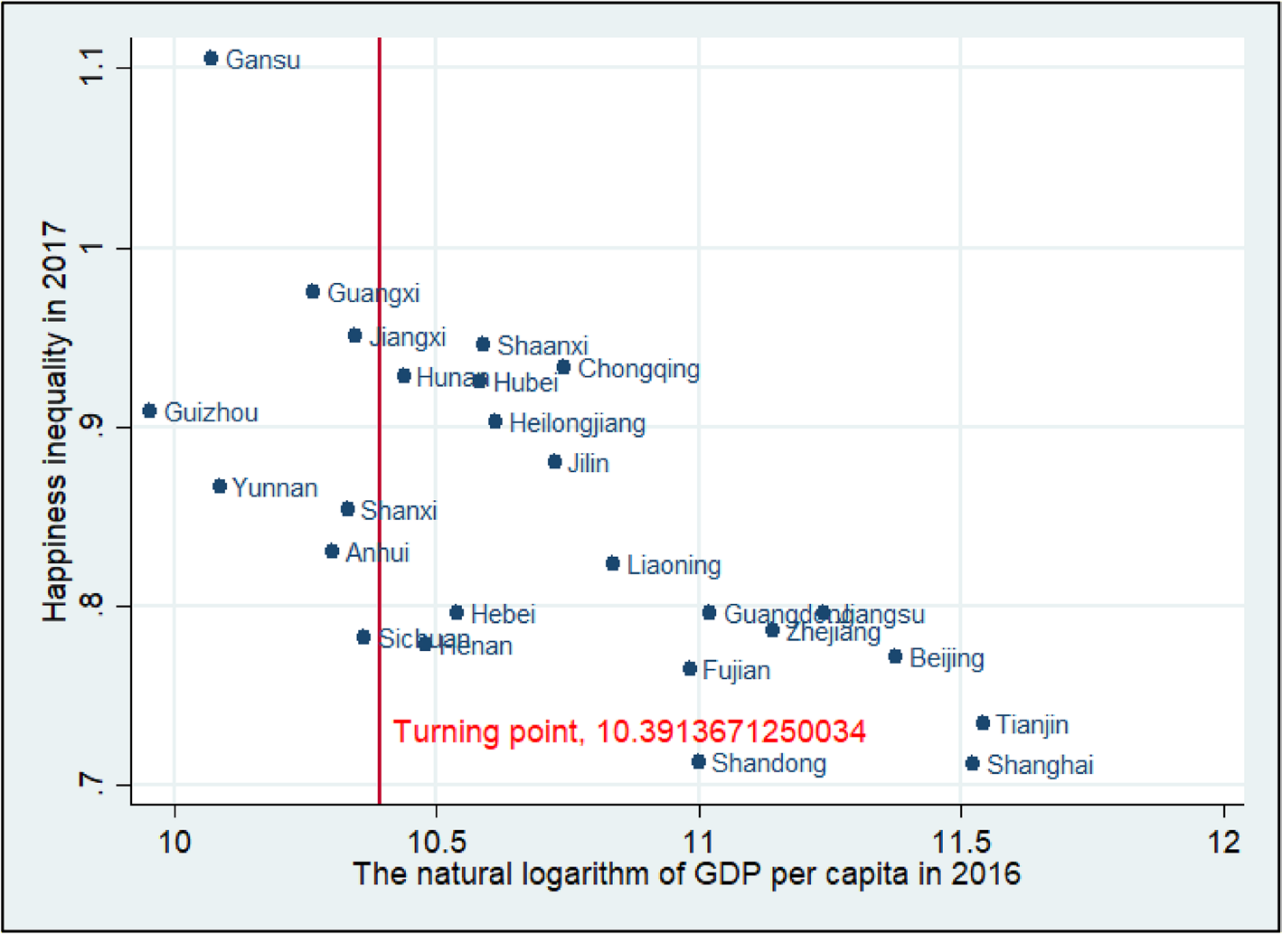
The turning point of the subjective wellbeing Kuznets curve, full sample^41,42^.

Figure 2 is a scatter plot based on happiness data from the 2017 CGSS. According to the estimation results for Model 1, the subjective wellbeing Kuznets curve hypothesis is supported and happiness inequality is highest when the natural logarithm of GDP per capita equals 10.3913671250034. Thus, a GDP per capita of about 32,577.17 Chinese yuan based on unchanged 2006 prices can be obtained from its natural logarithm, indicating that when the GDP per capita of one province is larger than 32,577.17 Chinese yuan based on unchanged 2006 prices, happiness inequality in this province is expected to decrease. This scatter plot shows that the GDP per capita of many provinces passed the turning point in 2016; these include Tianjin, Shanghai, Beijing, Jiangsu, Zhejiang, Guangdong, Shandong, Fujian, Liaoning, Chongqing, Jilin, Heilongjiang, Shaanxi, Hubei, Hebei, Henan, and Hunan.

## Discussion

### Theoretical contributions

Income inequality cannot capture all aspects of social inequality^4^, because income is a measure of life opportunities but fails to reflect life results^7,8^. Because subjective wellbeing measures individuals’ sense of quality of life and can figure as a good measure of life results, many scholars call for research into the inequality of subjective wellbeing^1,18,19,24^. Previous studies accumulate data about the antecedents of income inequality^5,44^, which can provide strong reference points for research on the inequality of subjective wellbeing. The Kuznets curve, which describes an inverted U-shaped relation between income inequality and economic growth, is an important hypothesis in the field of social inequality. Although many studies confirm the Kuznets curve in the context of income inequality^5^, no studies explore whether the Kuznets curve can describe the distribution of subjective wellbeing. Following previous research to use the standard deviation of people’s happiness to measure inequality of subjective wellbeing^17,20,26,27^, this study extends the Kuznets curve family to the study of the inequality of subjective wellbeing by examining the inverted U-shaped relationship between happiness inequality and economic growth.

The subjective wellbeing Kuznets curve can be understood from three perspectives. First, it is understandable that the relationship between happiness inequality and economic growth is an inverted U-shape, because income inequality shows a positive correlation with happiness inequality^4,8^ and income inequality has an inverted U-shaped relation with economic growth^5^. Second, economic growth can increase the quality of government, while both high technical quality of government and high democratic quality of government have inverted U-shaped relationships with happiness inequality^26,27^. From this perspective, the correlation between happiness inequality and economic growth should also have an inverted U shape. Third, the environmental Kuznets curve hypothesis^11,12^ and wealthier people’s stronger abilities to alleviate the negative effects of environmental pollution by investing in more effective air purifiers^34^ can also enhance the inverted U-shaped relationship between happiness inequality and economic growth.

### Policy implications

These results have several policy implications. Happiness inequality begins to decrease when per-capita GDP is around 32,577.17 Chinese yuan based on unchanged 2006 prices. Hence, to balance economic growth and happiness inequality, regions should strive to pass this turning point. However, because happiness inequality has an inverted U-shaped relation with economic growth, regional happiness inequality will increase continuously in regions at relatively low developmental stages. Meanwhile, high inequality is usually a source of social unrest and distrust in government. Thus, regions at a lower developmental stage should devote more effort to reducing happiness inequality as they develop their economies. Otherwise, social problems and high inequality may do fatal damage to social progress and government legitimacy.

Specifically, a series of policy measures can be used to control inequality. Some scholars find that the welfare state, as measured by decommodification measures and social wage measures, can reduce inequality in self-reported happiness^45^. Thus, welfare policies should be adopted and effectively implemented, such as a minimum wage policy, unemployment insurance plan, social medical insurance plan, and social assistance to disadvantaged groups. In addition, a certain level of government quality (technical quality of government and democratic quality of government) can help reduce happiness inequality^26,27^. Therefore, by improving government quality, it is possible to reduce happiness inequality, such as by improving government efficiency, controlling corruption, increasing people’s voices, and promoting the rule of law.

### Generalization and limitations

Though it examines the subjective wellbeing Kuznets curve, this study’s sample is limited to Chinese provinces. Thus, it is important to discuss the potential generalization of the empirical findings. It is likely that the inverted U-shaped relationship between happiness inequality and economic growth may hold in developing countries, particularly those in East Asia with stronger economic, geographic, and climatic similarities to China. However, it is less clear whether the subjective wellbeing Kuznets curve hypothesis can be extended to developed countries in Europe and North America. In addition, the dataset covers the period from 2003 to 2017. The subjective wellbeing Kuznets curve is more likely to be generalizable to other countries in this period than in other different periods. Thus, socio-economic conditions and time factors should be considered to generalize the empirical findings of this study.

This study has several limitations. First, it uses only data from Chinese provinces and fails to consider the potential influences of culture on people’s understanding of happiness. Thus, further study of a more global sample is warranted and samples from other countries can also be used to examine whether the subjective wellbeing Kuznets curve holds true in other cultures. Second, this study does not take limits to economic growth or planetary boundaries into consideration, while higher GDP may bring about ecological collapses. Future studies could examine the subjective wellbeing Kuznets curve using samples from countries at different development stages or regions with different ecological conditions. Third, happiness is measured using an ordinal 5-point Likert scale in CGSS and future studies can use an ordinal 11-point scale (0–10) to measure happiness, which can provide higher variance in happiness inequality. There are at least two questions left to be explored: whether the subjective wellbeing Kuznets curve exists in other parts of the world, and if so, whether these curves begin to turn at the same or a similar turning point. Answering these questions would deepen the understanding of the subjective wellbeing Kuznets curve.

## Conclusions

Using standard deviations of people’s happiness to measure inequality of subjective wellbeing, this study develops a panel data set based on ten waves of the CGSS and estimates a set of panel data models to examine the quadratic relationship between happiness inequality and economic growth. The results show that their relationship does follow a Kuznets curve, suggesting that as the economy grows, regional happiness inequality first increases, then reaches a peak, and finally decreases. Calculations show that happiness inequality in Chinese provinces begins to decrease when per-capita GDP is around 32,577.17 Chinese yuan based on unchanged 2006 prices. Comparing the turning point of the U-curve with the per-capita GDP of the sample provinces in 2016, this study finds that nearly two thirds of the 25 sample provinces had passed the turning point at that time, including Tianjin, Shanghai, Beijing, Jiangsu, Zhejiang, Guangdong, Shandong, Fujian, Liaoning, Chongqing, Jilin, Heilongjiang, Shaanxi, Hubei, Hebei, Henan, and Hunan.

## Supplementary Information

Supplementary Figures.

## Data Availability

Because of privacy or ethical restrictions, the data are only available on request from P. Z.
