# Supplementary material for: Happiness inequality has a Kuznets-style relation with economic growth in China
Source: Sci Rep. 2022 Sep 20;12:15712. doi: 10.1038/s41598-022-19881-3 (PMC9489784; doi:10.1038/s41598-022-19881-3)

Happiness inequality has a Kuznets-style relation with economic growth in China

Pan Zhang^1,2,*^

1. School of International and Public Affairs, Shanghai Jiao Tong University
2. China Institute for Urban Governance, Shanghai Jiao Tong University

Address: 1954# Huashan Road, Xuhui District, Shanghai, 200030

Email: zhang_pan@sjtu.edu.cn; Tel:(+86)18201828271

*Corresponding Author

**Data availability**

Because of privacy or ethical restrictions, the data are only available on request from P. Z.

**Acknowledgements**

This work was supported by the Shanghai Education Development Foundation and Shanghai Municipal Education Commission under Grant [number 19CG13]; National Natural Science Foundation of China under Grant [number 72074148].

**Author contributions**

P.Z. designed, wrote, and revised this paper.

**Competing interests**

The author declares no competing interests.

Supplementary Figure S1: Happiness Inequality in Chinese Provinces


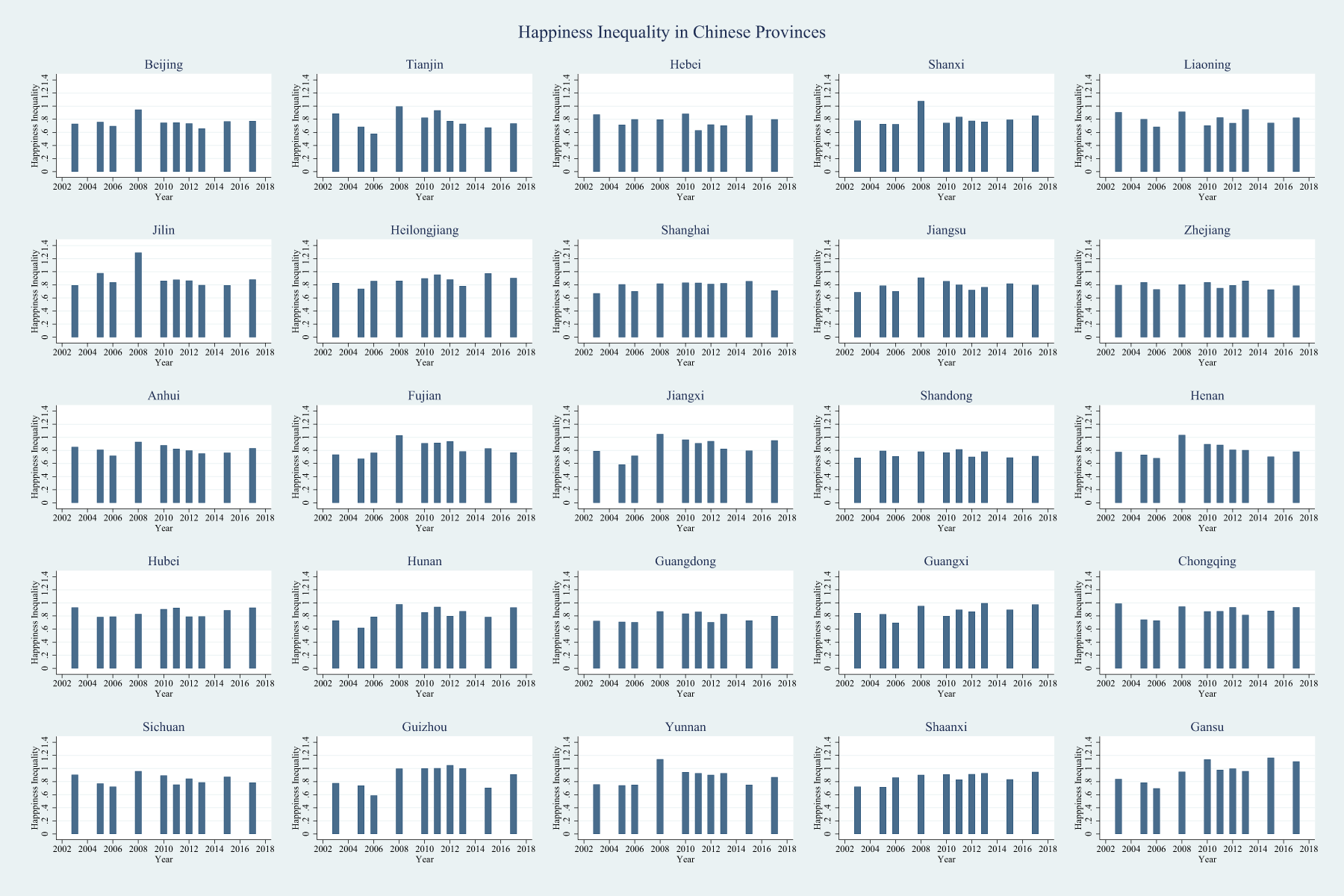


Supplementary Figure S2: GDP Per Capita in Chinese Provinces


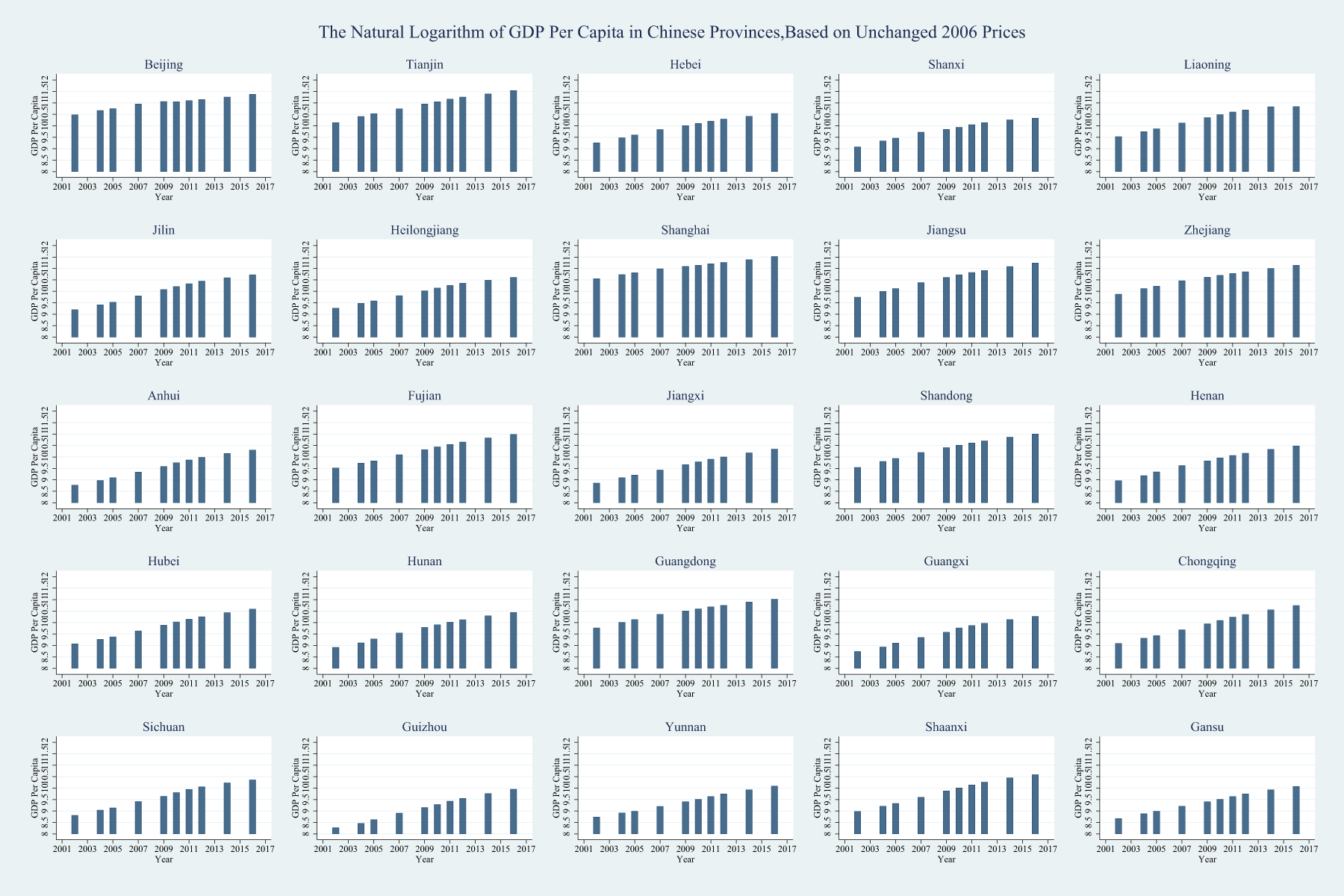

Supplement: Supplementary file 1 — Supplementary Figures. [file 41598_2022_19881_MOESM1_ESM.docx]
